# Pachymic acid alleviates hepatic lipid accumulation and oxidative stress in MASLD via regulation of the Keap1–Nrf2–HO-1 signaling axis

**DOI:** 10.3389/fnut.2026.1887176

**Published:** 2026-08-18

**Authors:** Maoru Li, Zefeng Wang, Huan Zhai, Jiajiao Li, Ji Ma, Bin Qiu

**Affiliations:** 1 Yunnan Key Laboratory of Chinese Medicine Processing, College of Chinese Materia Medica, Yunnan University of Chinese Medicine, Kunming, China; 2 School of Life Science and Technology, Kunming University of Science and Technology, Kunming, China

**Keywords:** antioxidant, Keap1–Nrf2–HO-1, MASLD, Pachymic acid, zebrafish

## Abstract

**Background:**

Metabolic dysfunction-associated steatotic liver disease (MASLD) is a rapidly growing metabolic disorder with no FDA-approved treatment. Pachymic acid (PA; synonym: 3-O-acetyltumulosic acid), a lanostane-type triterpenoid, shows potential for treating MASLD. However, its mechanisms remain underexplored.

**Objective:**

This work evaluated the therapeutic efficacy and mechanisms of PA in treating MASLD.

**Methods:**

The high-cholesterol diet (HCD)-induced zebrafish model, high-fat diet induced C57BL/6 mice model and free fatty acid (FFA)-treated BRL-3A cells was used to assess PA’s anti- MASLD effects *in vivo* and *in vitro*.

**Results:**

PA improved lipid metabolism disrupted by HCD, reduced hepatic oxidative stress, as indicated by decreased MDA levels and increased SOD activity, and lowered inflammatory markers, including TNF-*α*, IL-6, and IL-1β. PA also enhanced antioxidant activity by regulating the Keap1-Nrf2-HO-1 pathway. PA decreased Keap1 mRNA expression induced by reactive oxygen species (ROS), upregulated Nrf2 and HO-1 expression, and promoted Nrf2 nuclear translocation. These effects suggest that PA inhibits ROS production by activating the Keap1-Nrf2-HO-1 pathway and possibly by inhibiting Nrf2 degradation.

**Conclusion:**

PA improves MASLD through lipid metabolism regulation and antioxidant effects, primarily by activating the Keap1-Nrf2-HO-1 pathway. These findings highlight PA’s potential as a therapeutic agent for MASLD.

## 1 Introduction

Nonalcoholic fatty liver disease (NAFLD) was initially characterized in the 1990s. It has become one of the fastest-growing clinical metabolic syndromes, with an estimated global prevalence of 32% in adults (1). The primary lesion types in NAFLD are hepatic steatosis (also referred to as nonalcoholic fatty liver) and nonalcoholic steatohepatitis (NASH). These are distinguished by hepatocyte steatosis, lipid oxidation, inflammation, and the potential progression to fibrosis and cirrhosis (2, 3). Recently, experts have put forward “metabolic dysfunction-associated steatotic liver disease (MASLD)” as an alternative term for NAFLD, considering the major role metabolic dysfunction plays in the syndrome (4). However, no FDA-approved therapeutic drugs for MASLD/NASH are available since a comprehensive understanding of the multiple pathogenesis factors is lacking (5).

The classic “two-hit” theory explains the development of MASLD through two sequential events: initial hepatic steatosis due to lipid overload, followed by inflammation triggered by oxidative stress (6). However, with advances in knowledge from new studies, the “two-hit” theory’s explanations for the intricate pathogenesis of MASLD are deemed inadequate. Consequently, a novel “multiple-hit” concept has surfaced, encompassing factors such as insulin resistance, lipid accumulation, oxidative stress, inflammation, adipokines, bile acids, and others (7). However, these two theories share a common understanding of the central roles played by lipid accumulation and oxidative stress. Therefore, lipid-lowering and antioxidant compounds are considered beneficial in treating MASLD. Dietary intervention has long been acknowledged as an effective approach to prevent and manage MASLD. Furthermore, numerous studies have documented the effects of medicinal and edible plants on MASLD (8, 9). Therefore, developing functional foods to treat MASLD is of great significance.

*Poria cocos*, an edible fungus (10), has an annual demand of 10,000–13,000 metric tons in Asia. It has been used in traditional Chinese medicine for millennia, where it is commonly prescribed in formulations utilizing its purported antioxidant and anti-inflammatory properties (11). Guizhi Fuling Wan, first documented in the Jin Gui Yao Lue (Synopsis of the Golden Chamber) by Zhang Zhongjing during the Eastern Han Dynasty, is traditionally used to promote blood circulation and resolve blood stasis. Similarly, Linggui Zhugan Tang, which is also derived from the same classical text, is used to warm yang and resolve dampness. Modern studies have demonstrated that both formulas exert regulatory effects on glucose and lipid metabolism (12, 13). Notably, Zhu Ling Tang, another classical formula documented in the Shang Han Lun, has shown significant antioxidant activity (14). Existing research indicates that the bioactive components of *Poria cocos* predominantly comprise triterpenoids and polysaccharides (15).

Pachymic acid (PA; synonym: 3-O-acetyltumulosic acid) is a major bioactive lanostane-type triterpenoid constituent of *Poria cocos* (16). PA has been extensively documented for its notable anti-inflammatory impact on local inflammation in the skin, oral cavity, and the ear canal (17). This effect is mediated by the suppression of phospholipase A2 (PLA2) and leukotriene B4, and the reduced functions of 5-lipoxygenase and COX-2 (18). Recent studies have indicated that PA effectively alleviates lipid metabolism disorders (19). Additionally, PA inhibits NF-κB translocation while enhancing NRF2 translocation, leading to increased HO-1 protein levels and alleviation of H_2_O_2_-induced inflammation and oxidative stress (20). However, research on PA as a potential therapeutic agent for MASLD is limited (21).

Further research is required to unravel the molecular mechanisms underlying PA’s anti-MASLD properties. Therefore, we fed zebrafish larvae a high-cholesterol diet (HCD), C57BL/6 mice a high-fat diet (HFD) and treated BRL-3A cells with FFA to generate models of MASLD. These models were employed to examine the lipid-metabolism-regulating, antioxidant, and anti-inflammatory effects of PA *in vivo* and *in vitro*. Knowledge generated on how PA mitigates MASLD will help develop drugs and functional diets for MASLD management.

## 2 Materials and methods

### 2.1 Reagents and solutions

PA (analytical standard, >99%; National Institutes for Food and Drug Control (NIFDC), Beijing, China); Nile red (95%) and pioglitazone (PIO; 98%), an established lipid-regulating drug (Shanghai Aladdin, China); cholesterol (92.5%), oil red O (ORO; 98%), and DCFH-DA (Sigma-Aldrich, USA); fetal bovine serum (FBS) and Dulbecco’s Modified Eagle’s Medium (DMEM; Gibco, USA).

### 2.2 PA toxicity assessment in zebrafish

Wild-type zebrafish (AB strain) were raised in recirculating housing systems at 28 °C under a 14/10 (light/dark) photoperiod. Embryos generated through natural mating were collected. PA toxicity was assessed using zebrafish embryo acute toxicity test methods A and B (T/GDST 1–2021). Four hours after fertilization, zebrafish embryos were individually exposed to test solutions containing various concentrations of PA (2.5, 5, 10, 20, and 40 μM; *n* = 30 per group) or solvent controls in 6-well plates. The semi-static test included daily renewal of the solutions from 24 to 96 h post-fertilization (hpf) and daily measurement of zebrafish body length. Hatching rates and cumulative mortality were assessed upon completion of the experiment.

### 2.3 Maintenance of zebrafish and treatments

Embryos produced through natural mating were collected and reared in egg water at 28 °C under a 14/10 (light/dark) photoperiod until 5 dpf. Subsequently, the larvae were randomly allocated into five experimental groups (*n* = 200 per group): (1) control group (Control), larvae fed a normal diet; (2) model group (HCD), larvae fed a HCD diet; (3) PIO administration group (PIO) as positive control, larvae fed an HCD and treated with PIO; (4) Low-dose PA group (PAL); and (5) High-dose PA group (PAH), larvae fed an HCD and treated with PA.

Zebrafish larvae were fed an HCD to induce MASLD, following an established protocol (22). The HCD was prepared by supplementing the basic food (Gardners, PA, USA) with 5% (w/w) cholesterol. The other compounds were prepared as follows: PIO (a selective PPARγ agonist that improves insulin sensitivity and hepatic histology in patients with MASLD) served as a positive control to validate our screening approach (23). PIO was prepared as a DMSO stock due to its low water solubility and then diluted with water to a working concentration of 1 μM (0.001% DMSO, v/v); PA was dissolved in water to yield 5 μM (PAL) and 10 μM (PAH) solutions. DMSO (0.001%, v/v) was added to exclude the effect of DMSO in the zebrafish experiment.

### 2.4 Maintenance of mice and treatments

High-Fat-Diet (HFD) diet (XTHF45) and normal diet (ND, XTCON50H) were purchased from Xietong Bio Co. LTD (Nanjing, China). 48 male C57BL/6 mice (18 to 22 g) were purchased from SPF (Beijing) biotechnology co., Ltd. After administration with HFD or ND for 10 weeks, mice were randomly divided into 4 groups (*n* = 12) as follows: Control group fed with ND and vehicle (Saline); Model group fed with HFD diet and vehicle (Saline); PA group fed HFD and PA (10 mg/kg/day, preliminary experiments have shown that, at a dose of 10 mg/kg, PA exhibits the most significant lipid-lowering and antioxidant effects); PIO group (PIO) administered HFD and PIO (10 mg/kg/day) (24).

### 2.5 BRL-3A cell viability evaluation and experimental treatments

BRL-3A cells (Rattus norvegicus hepatocellular lineage; ATCC, Manassas, VA, USA) were cultured in complete DMEM supplemented with 10% FBS at 37 °C and 5% CO_2_. The optimal PA concentration for subsequent experiments was screened using the CCK-8 assay. Cells were seeded at 5 × 10^4^ cells/cm^2^ in 6-well plates. Upon reaching 70–80% confluence, cells were allocated to four groups for a 24-h treatment: (1) control, a blank solvent without FFA; (2) model, supplemented with a 1 mM FFA cocktail composed of oleic acid and palmitic acid at a 2:1 ratio; (3) PA, supplemented with a 1 mM FFA cocktail plus 20 μM PA; and (4) PIO, supplemented with a 1 mM FFA cocktail plus 1 μM PIO. Experiments were conducted in triplicate.

Additionally, ML-385(5 μM; HY-100523, MedChemExpress), a specific NRF2 inhibitor, was used to inhibit NRF2 in BRL-3A cells. In this case, the experimental groups were defined as follows: (1) control, vehicle only without FFA; (2) ML-385, supplemented with a 1 mM FFA cocktail plus 5 μM ML-385; and (3) ML-385 + PA, supplemented with a 1 mM FFA cocktail plus 5 μM ML-385 and 20 μM PA. Treatment outcomes were assessed after 24 h.

### 2.6 ORO staining and histopathology

ORO staining was performed as described in a previous study (25). A working ORO solution was prepared by diluting the stock solution (0.5 mg/mL in isopropyl alcohol) with water at a 3:2 (v/v) ratio, followed by filtration until clear. Zebrafish larvae were fasted for 24 h and then anesthetized with 0.05% tricaine. The anesthetized zebrafish larvae were fixed in 4% paraformaldehyde at 4 °C approximately 48 h, washed twice with PBS, and sequentially infiltrated by incubation in increasing concentrations of 1,2-propylene glycol for 20 min each. Subsequently, the larvae were stained with 0.5% ORO in the dark for 24 h. The stained larvae underwent two additional 2-min washes with PBS. Zebrafish specimens were examined and imaged using an Olympus stereomicroscope (Tokyo, Japan). The ORO staining intensity in the acquired images was quantified using ImageJ software.

The zebrafish blocks and liver tissues were fixed in 4% paraformaldehyde for 24 h at room temperature, then paraffin-embedded and sectioned at 4 μm. After washed with a gradient con centration of ethanol solution for hematoxylin–eosin (HE) staining, dehydration and neutral resin sealing. The prepared tissue sections were observed under a stereoscope (Olympus, Tokyo, Japan). All samples stained with H&E according to standard procedures (26, 27).

### 2.7 Fluorescence imaging

Nile red (*n* = 10 per group) and DCFH-DA (*n* = 10 per group) staining were used in this study. Nile red is a lipid fluorescent dye that labels neutral lipids with bright red fluorescence. Nile red fluorescence was detected at excitation/emission (Ex/Em) wavelengths of 543/598 nm. DCFH-DA is a reactive oxygen species (ROS) indicator that is oxidized by ROS in living cells to produce 2′,7′-dichlorofluorescein (DCF), which is detected at Ex/Em wavelengths of 480/525 nm. After PA treatment, BRL-3A cells were incubated with Nile red (0.5 μg/mL) at 37 °C for 30 min and imaged using a Nikon Eclipse Ni-U fluorescence microscope. Zebrafish larvae were incubated for 30 min at 28.5 °C in the dark with either Nile red (0.5 μg/mL) or DCFH-DA (10 μM), rinsed three times with egg water, anesthetized with 0.05% tricaine, and embedded in 4% carboxymethyl cellulose sodium (CMC-Na) for imaging. Images were acquired using a fluorescence stereoscope (SZX16, Olympus).

### 2.8 Biochemical measurements

Biochemical indices, including triglycerides (TG), total cholesterol (TC), superoxide dismutase (SOD), and malondialdehyde (MDA), were analyzed using commercially available assay kits (Jiancheng, Nanjing, China) according to the manufacturer’s instructions. ROS levels were quantified using a BeyoTime kit (China) according to the manufacturer’s protocol. HO-1 levels were determined using an HO-1 ELISA kit (Jiancheng). Sample absorbance was recorded using a multimode microplate reader (BioTek, USA).

### 2.9 Immunofluorescence staining of BRL-3A cells

BRL-3A cells were processed for immunofluorescence staining after a 24-h exposure to FFA cocktail with or without PA. Cells were washed twice with PBS, fixed in 4% paraformaldehyde on ice for 15 min, blocked for 1 h at room temperature in 5% BSA containing 0.3% Triton X-100, and incubated overnight at 4 °C with anti-NRF2 primary antibody (1:200; Invitrogen). Subsequently, cells were incubated with an FITC-conjugated secondary antibody (37 °C, 20 min) and then counterstained with DAPI. Images were acquired using a fluorescence microscope.

### 2.10 Reverse transcription real-time quantitative PCR (RT-qPCR) analysis

Total RNA was extracted from pooled samples of 30 treated zebrafish larvae per group or from BRL-3A cells using TRIzol reagent (Invitrogen, Waltham, MA, USA). Total RNA was reverse transcribed into cDNA using a commercial kit. SYBR Green Master Mix (Takara, Japan) was used for qPCR according to the manufacturer’s protocol. Relative transcript levels were calculated after normalization to GAPDH as the internal control. Primer sequences are provided in Supplementary Table S1.

### 2.11 Antibodies and western blot (WB)

Total protein was isolated using RIPA buffer supplemented with protease inhibitors. Protein samples (equal loading) were separated on 4–12% gradient gels by SDS–PAGE, transferred onto PVDF membranes, and incubated overnight at 4 °C with an-ti-NRF2 (1:1,000; #20733), anti-HO-1 (1:1,000; #43966), anti-*β*-actin (1:1,000; #4967), and anti-lamin B1 (1:1,000; #13435) antibodies. After washing, the membranes were incubated with a secondary antibody (1:5,000; #7074) for 1 h at room temperature. Bands were detected using an ECL chemiluminescence detection kit (ProteinTech Group, Chicago, IL, USA) and quantified using ImageJ (version 1.53).

### 2.12 Statistical analysis

Continuous variables are expressed as mean ± SD based on at least three independent replicates. Statistical analysis was performed using GraphPad Prism 9.5.0 (San Diego, CA, USA). Group comparisons were conducted using one-way ANOVA followed by Dunnett’s multiple-comparisons test. Statistical significance was set at *p* < 0.05.

## 3 Results

### 3.1 Safety assessment of PA in zebrafish larvae

**Figure 1:**
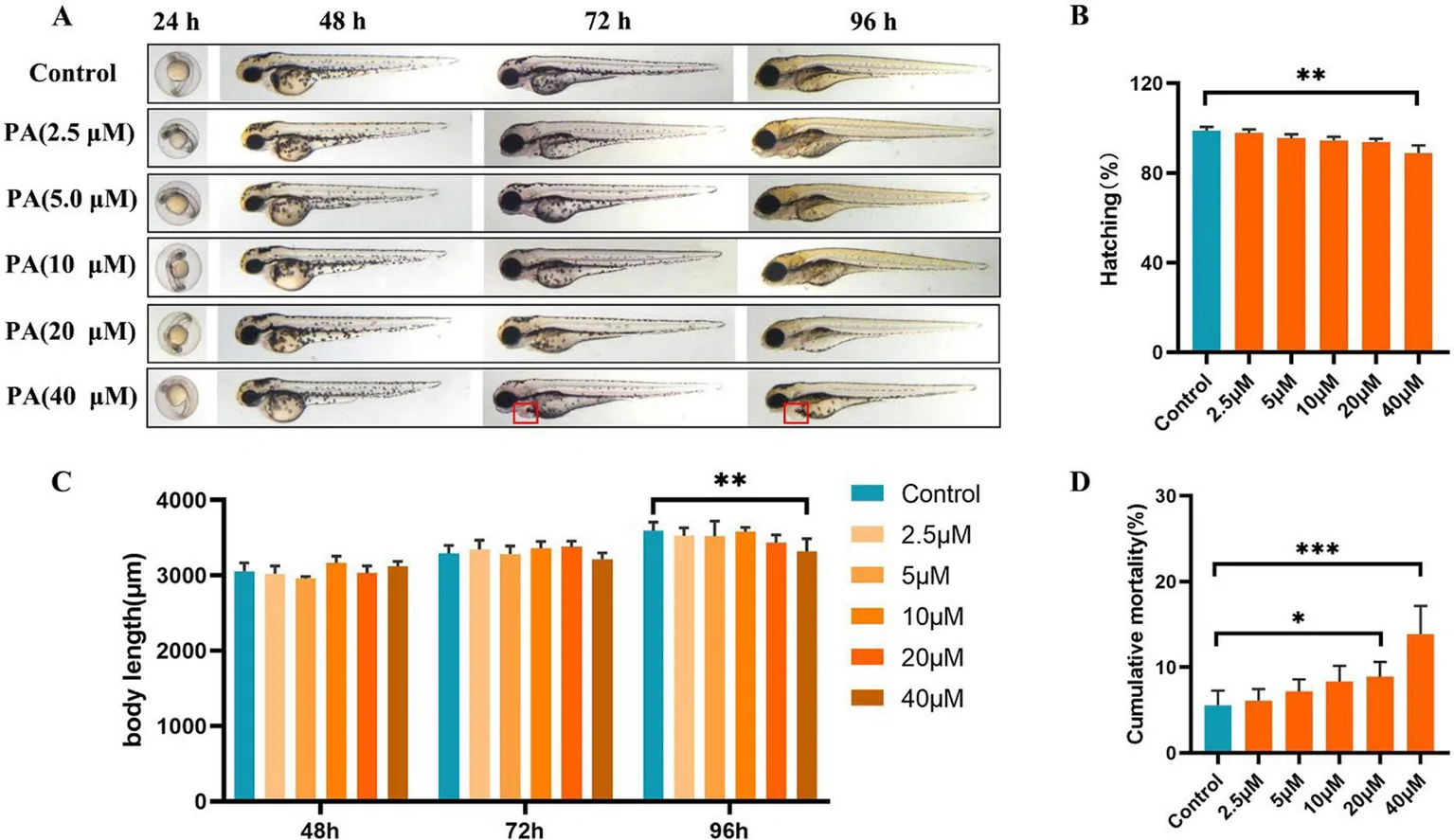
PA toxicity assessment in zebrafish larvae exposed to concentrations of 2.5–40 μM (*n* = 30). **(A)** Representative images of zebrafish embryos and larvae at 24, 48, 72, and 96 hpf. Pericardial edema is marked by a red border. Effect of PA exposure on **(B)** hatching rate, **(C)** body length, and **(D)** cumulative mortality. Bars represent means ± SD. **p < 0.01 and ***p < 0.001 compared with the control group. Group means were compared using one-way ANOVA followed by Dunnett’s multiple-comparisons test. Experiments were performed in triplicate.

The initial experiment assessed the dose-dependent effects of PA on key physiological parameters in zebrafish embryos. A significant decrease in hatching rates (*p* = 0.0012, Figure 1B) and an increase in cumulative mortality (*p* < 0.0001, Figure 1D) were observed at a PA concentration of 40 μM. Pericardial effusion was observed beginning at 72 hpf (Figure 1A), and growth was significantly inhibited at 96 hpf (*p* = 0.0053, Figure 1C). These results suggest a safe PA concentration range of up to 10 μM under our experimental conditions.

### 3.2 PA alleviates HCD-induced MASLD in zebrafish larvae

**Figure 2:**
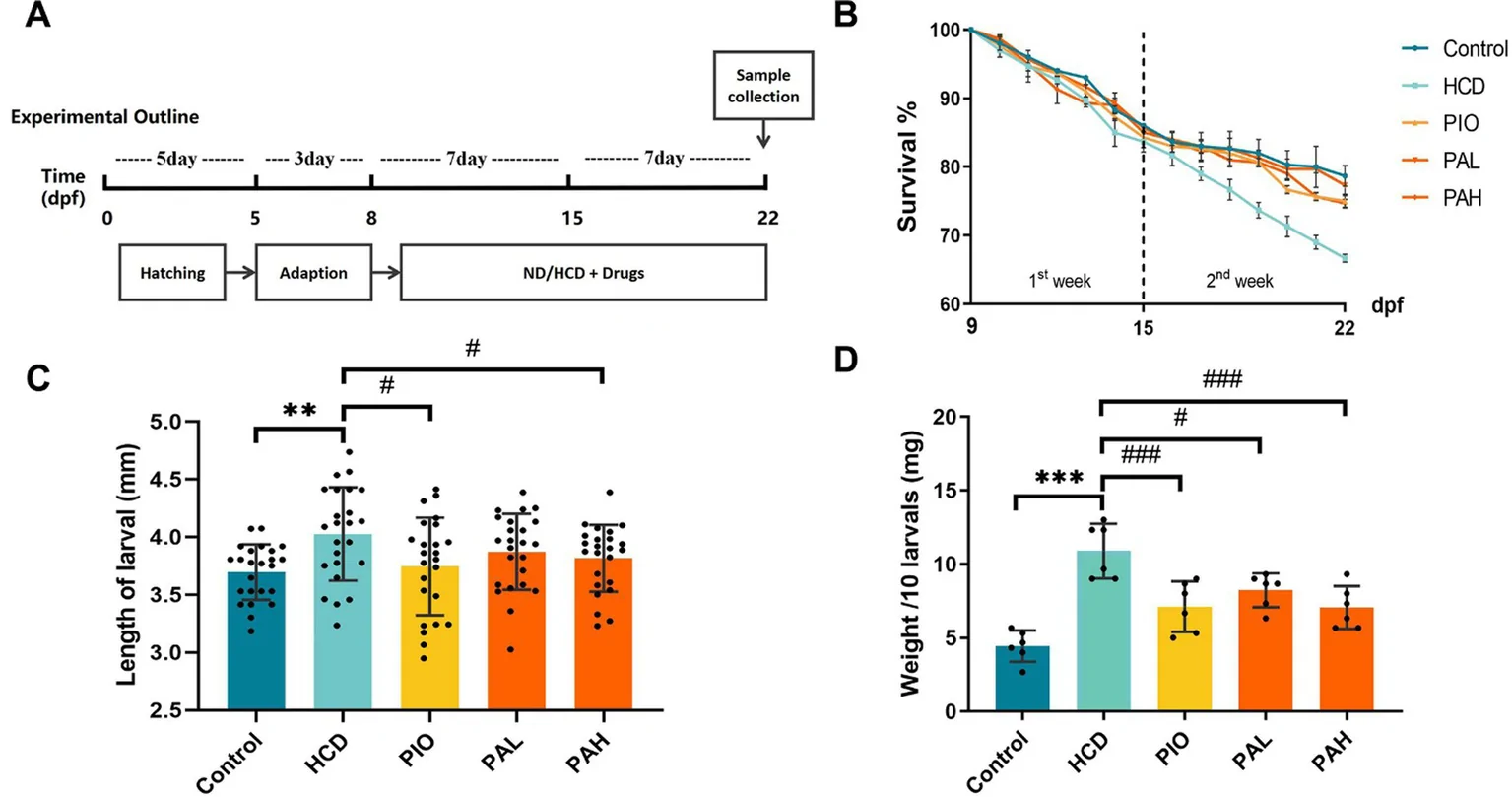
Effects of PA on the HCD-induced MASLD model in zebrafish larvae. **(A)** Schematic overview of the *in vivo* study design. **(B)** Survival rate of zebrafish larvae. **(C)** Body length (*n* = 25). **(D)** Body weight (*n* = 6). Bars represent means ± SD. **p < 0.01 and ***p < 0.001 compared with the control group. Group means were compared using one-way ANOVA followed by Dunnett’s multiple-comparisons test. Experiments were performed in triplicate.

Larval zebrafish were administered from 6 dpf to 21 dpf following the schedule presented in Figure 2A. The effects of PA on HCD-fed zebrafish larvae were assessed by survival rate, body length, and body weight after 14 days of HCD feeding (Figures 2B–D). Body length (*p* = 0.0051) and body weight (*p* < 0.0001) in the HCD group were significantly greater than in the control group (Figures 2C,D), confirming the validity of the experimental model. Moreover, body weight in the HCD group was significantly higher than in the PA and PIO groups (both *p* = 0.0012), as shown in Figure 2C. Body length and body weight decreased in a dose-dependent manner in the PA-treated groups (PAL and PAH). Survival rates in both the PA and PIO groups were significantly higher than in the HCD group (Figure 2B). These results demonstrate the pharmacological activity of PA in the HCD-induced MASLD model.

**Figure 3:**
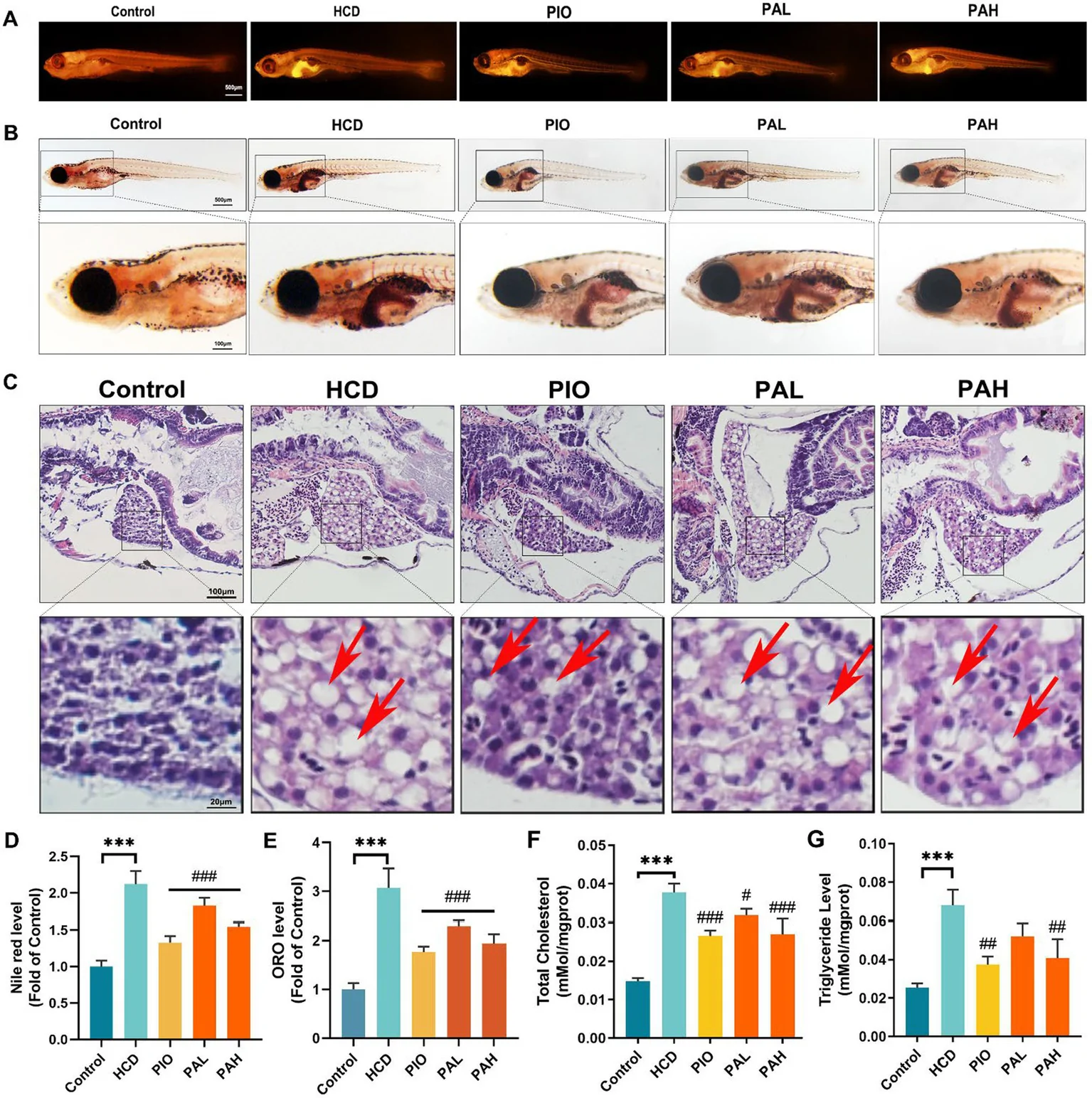
Effects of PA on lipid metabolism in the HCD-induced MASLD model in zebrafish larvae. **(A)** Nile red staining (*n* = 10). **(B)** ORO staining (*n* = 10). **(C)** H&E staining of zebrafish larval livers (macrovesicular steatosis marked with a red arrow; *n* = 10). **(D)** Quantitation of Nile red stain. **(E)** Quantitation of Oil Red stain. **(F)** TC levels (*n* = 18). **(G)** TG levels (*n* = 18). Bars represent means ± SD. ***p < 0.001 vs. control; ###*p* < 0.001 vs. model. Statistical signif-icance was set at *p* < 0.05. Group means were compared using one-way ANOVA followed by Dunnett’s multiple-comparisons test. Experiments were performed in triplicate.

Zebrafish larvae from the five experimental groups were stained with Nile red (22), and the fluorescence intensity of each fish was measured to assess the effects of PA on lipid metabolism regulation. As shown in Figures 3A,D, lipid accumulation was markedly higher in the HCD group than in the control, PA, and PIO groups. ORO is a lipophilic dye that stains neutral lipids orange-red (28). Whole-body ORO staining was performed on zebrafish larvae to visualize lipid accumulation (Figure 3B). ORO staining showed markedly increased lipid deposition in the abdominal region of HCD-fed larvae, mainly corresponding to the liver area. In contrast, PA treatment (PAL and PAH) and PIO treatment substantially reduced ORO-positive staining compared with the HCD group, indicating alleviation of hepatic steatosis (Figure 3E).

Histopathological sections (Figure 3C) provided direct evidence of lipid accumulation and metabolic alterations among the five experimental groups. Macrovesicular steatosis levels in the PA and PIO groups were significantly lower than in the HCD group. Quantification of TG and TC levels revealed significant lipid accumulation in the HCD group compared with all other groups (Figures 3F,G). Moreover, the lipid-lowering effect of PA was concentration-dependent. These findings indicate that PA exerts a lipid-regulating effect in a zebrafish larval model of HCD-induced MASLD.

**Figure 4:**
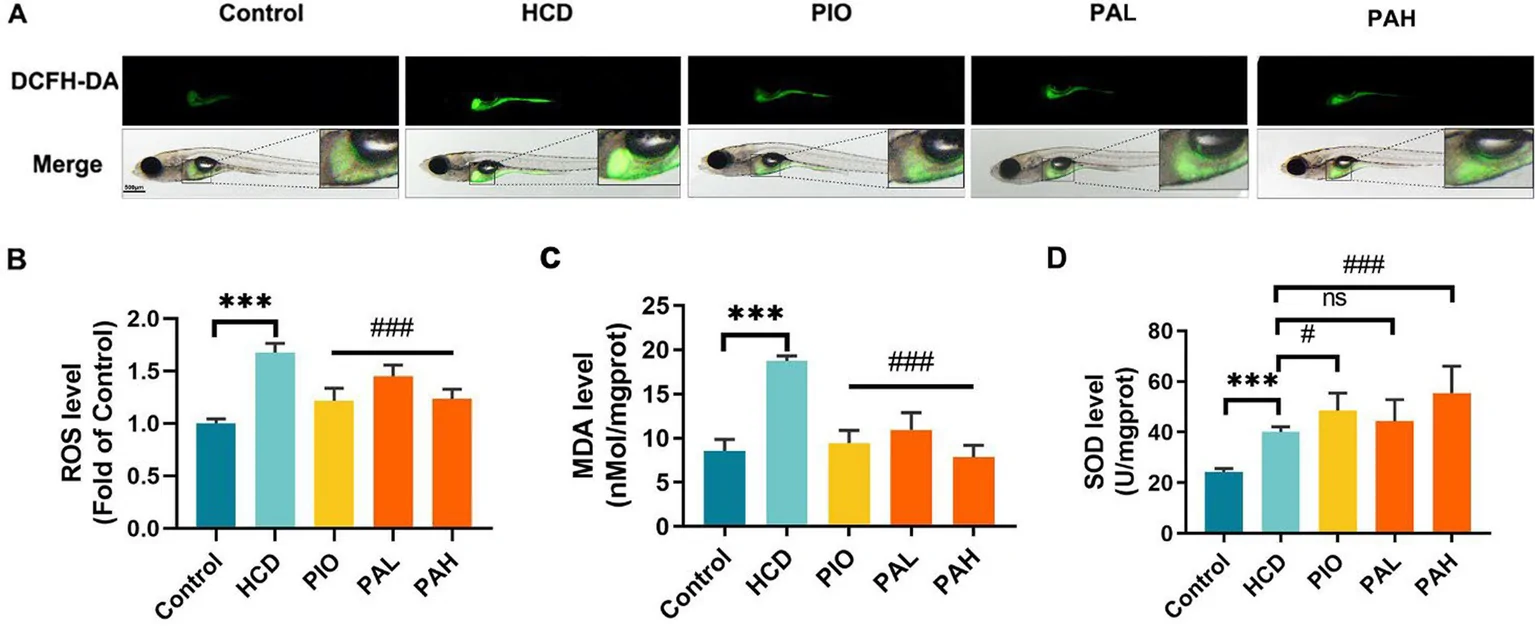
Effects of PA on oxidative stress in the HCD-induced MASLD model in zebrafish larvae. **(A)** ROS levels detected by DCFH-DA staining (*n* = 10). **(B)** Quantitative ROS analysis (*n* = 10). **(C)** SOD activity (*n* = 18). **(D)** MDA levels (*n* = 18). Bars represent means ± SD. ***p < 0.001 vs. control; ###*p* < 0.001 vs. model. Statistical significance was set at *p* < 0.05. Group means were compared using one-way ANOVA followed by Dunnett’s multiple-comparisons test. Experiments were per-formed in triplicate.

To explore PA’s influence on subsequent lipid metabolism, we evaluated oxidative stress in zebrafish larvae through DCFH-DA analysis, a fluorescent stain used to assess the ROS level. The staining results (Figure 4A) showed higher ROS levels in the larval abdomen of the HCD group relative to the control, PA, and PIO groups. To delineate the antioxidant effect of PA in HCD-fed zebrafish larvae, we assessed substances involved in the oxidation pathway, including the downstream products of lipid oxidation (MDA and ROS) together with the activity of superoxide dismutase (SOD). The findings (Figures 4B,C) indicated lower levels of ROS (*p* < 0.0001) and MDA (*p* < 0.0001) in the PA group compared to the model group, while its SOD activity was higher (PAH, *p* < 0.0001, Figure 4D). These results indicate that PA, like PIO, exerts an antisteatotic effects through antioxidative mechanisms.

### 3.3 PA changes mRNA expression in the zebrafish larval model *in vivo*

Based on the pharmacodynamic evaluation, the PAH group showed the strongest lipid-regulating and antioxidant effects among the PA-treated groups and was therefore selected for subsequent mechanistic analysis. To further elucidate the mechanisms underlying the effects of PA on MASLD pathogenesis, RT-qPCR was performed to evaluate mRNA expression related to inflammation, fibrosis, lipid metabolism, and oxidative stress.

**Figure 5:**
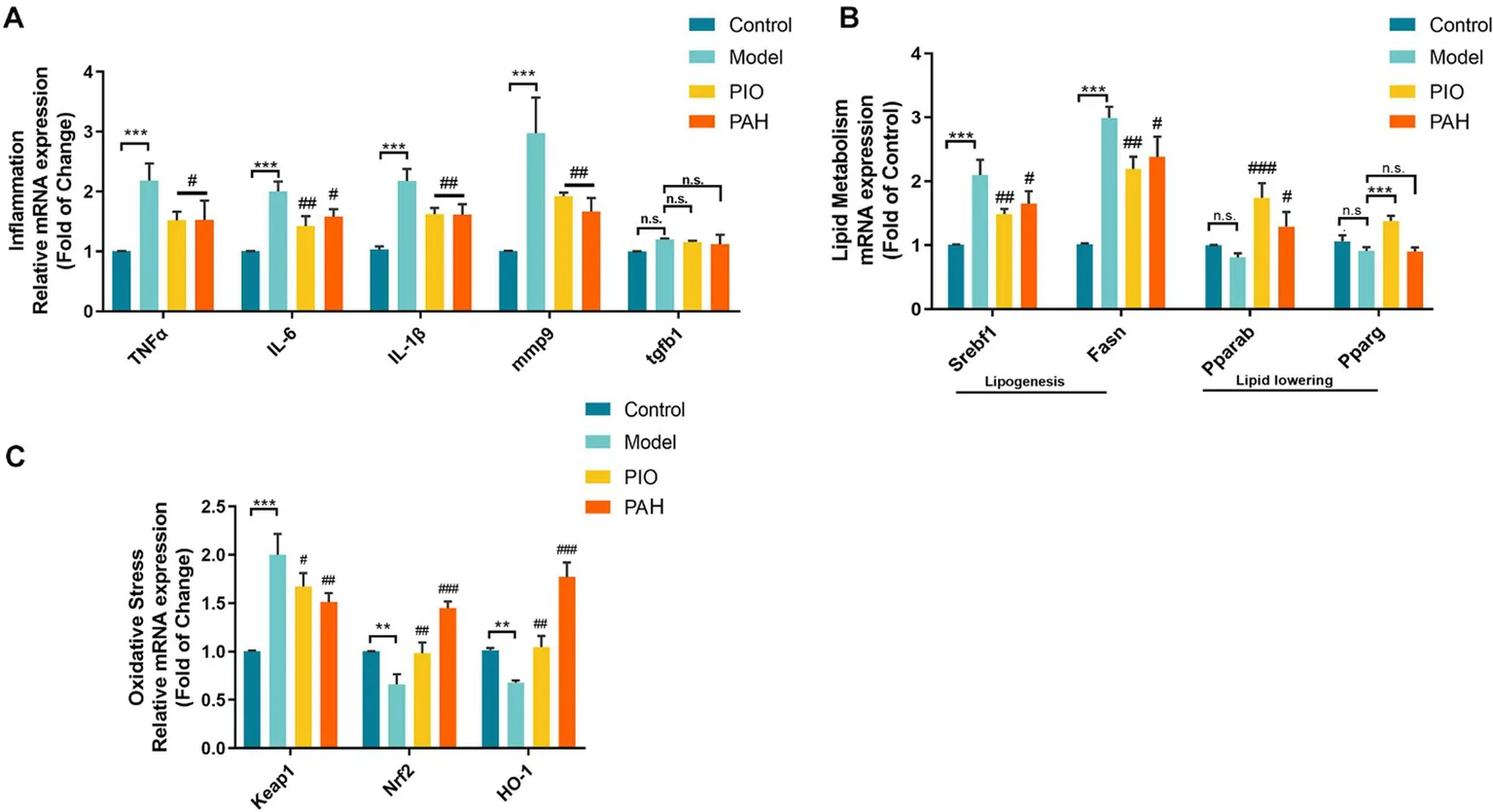
PA-induced changes in mRNA expression in zebrafish larvae (*n* = 30). **(A)** Inflammation-related gene expression. **(B)** Gene expression associated with lipid metabolism. **(C)** Oxidative stress-related gene expression. Bars represent means ± SD. n.s.: Not significant. *p < 0.05, **p < 0.01, and ***p < 0.001 vs. control; #*p* < 0.05, ##*p* < 0.01, and ###*p* < 0.001 vs. HCD group. Statistical significance was set at *p* < 0.05. Group means were compared using one-way ANOVA followed by Dunnett’s multiple-comparisons test. Experiments were performed in triplicate.

The mRNA expression of key pro-inflammatory mediators, including interleukin 6 (il6), interleukin 1β (*il1b*), tumor necrosis factor alpha (*tnf-α*), transforming growth factor β1 (*tgfb1*), and matrix metalloproteinase 9 (*mmp9*), was analyzed (Figure 5A). Among these, *mmp9* and *tgfb1* are closely associated with liver fibrosis. PA treatment significantly decreased the mRNA expression levels of *mmp9* (*p* = 0.0026), *il1b* (*p* = 0.0039), *tnfa* (*p* = 0.0183), and *il6* (*p* = 0.0121) compared with the HCD group. Similar effects were observed in the PIO group. In contrast, *tgfb1* mRNA expression did not differ significantly among the four experimental groups. These results suggest that PA exerts its anti-inflammatory effects by suppressing key inflammatory mediators at the transcriptional level.

Compared with the HCD group, the PA group showed significantly reduced transcript levels of the lipogenic genes sterol regulatory element binding proteins (*srebf1*; *p* = 0.0222) and fatty acid synthase (*fasn*; *p* = 0.0156) (Figure 5B). Genes involved in lipid metabolism, including peroxisome proliferator-activated receptor alpha (*ppara*) and gamma (*pparg*), were also assessed. *Ppara* expression in the PA (*p* = 0.019) and PIO (*p* = 0.0004) groups (Figure 5B), and *pparg* expression in the PIO group (*p* = 0.0002) were significantly increased compared with the HCD group. In contrast, the expression levels of *pparg* in the PA and HCD groups were statistically comparable (Figure 5B). These findings indicate that PA modulates lipid metabolism by regulating the mRNA expression levels of *srebp1*, *fasn*, and *ppara*.

Expression of kelch-like ECH-associated protein 1 (*keap1*) in the PA (*p* = 0.006) and control (*p* < 0.0001) groups was lower compared with the HCD group (Figure 5C). Both the nuclear factor erythroid 2-related factor 2 (*Nrf2*; *p* < 0.0001), responsible for activating the antioxidative pathway, and its downstream antioxidant effector HO-1 (*p* < 0.0001) were significantly upregulated in the PA group compared with the HCD group. The PA group exhibited greater effects than the PIO group, indicating that PA is important in enhancing antioxidative activity.

### 3.4 Effect of PA on an HFD-induced mice MASLD model *in vivo*

**Figure 6:**
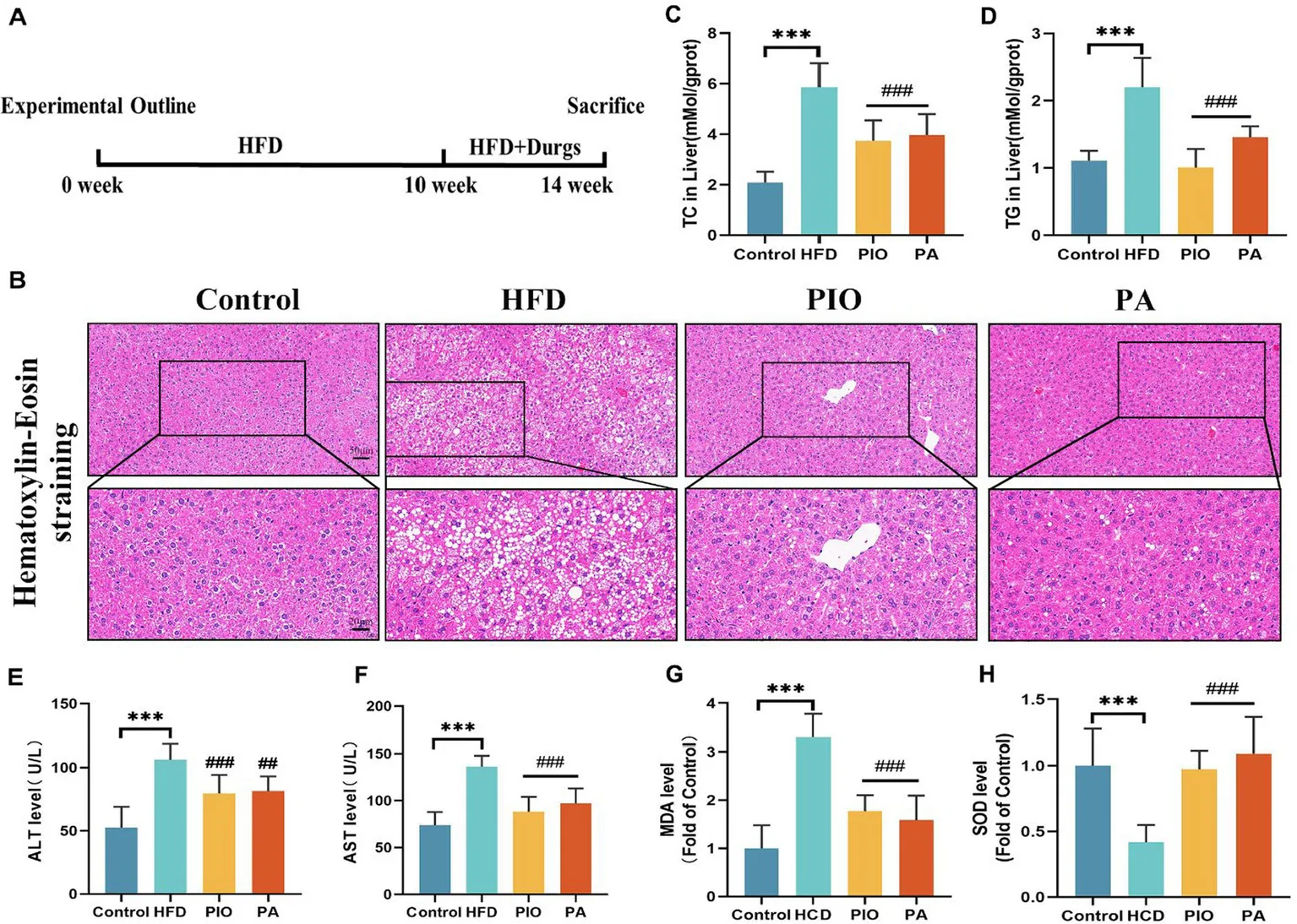
Effect of PA on an HFD-induced mice MASLD model in vivo. **(A)** Experimental outline of the mice experiments in vivo. **(B)** Hematoxylin and eosin (HE) staining of mice liver. **(C)** Total cholesterol (TC, mmol/mg prot) of mice liver. **(D)** Triglyceride (TG, mmol/mg prot) of mice liver. **(E)** ALT level of mice serum. **(F)** AST level of mice serum. **(G)** MDA level of mice liver. **(H)** SOD level of mice liver. Bars represent means ± SD. ***p < 0.001 vs. control; ###*p* < 0.001 vs. model. *p* < 0.05 was considered statistically significant calculated by One-way ANOVA followed by Dunnett’s multiple-comparisons test. (n = 6 for B; n = 12 for C-H).

To verify the therapeutic effect of PA on MASLD, a High-Fat diet (HFD) induced mice model was used and PIO, a well-known lipid regulating drug, is used as a positive control. The experimental flowchart is shown in Figure 6A. To reveal the pathological changes in the liver, hematoxylin–eosin (HE) stain was performed on all four groups of mice, which could identify the steatosis of the liver. From the result of HE stains we can see (Figure 6B), that macrovesicular steatosis can be widely found in the HFD group, which indicates that a successful MASLD model was built. Both PIO and PA reversed the macrovesicular compared to the HFD group. Biochemical measurements of hepatic triglyceride (TG) and total cholesterol (TC) further show the lipid accumulation of the four groups. Results (Figures 6C,D) proved the positive lowering lipid accumulation effects of both PIO (*p* < 0.0001) and PA (*p* < 0.0001) compared to the model group on the HFD mice model. A conclusion can be drawn that PA has a lipid-regulating effect on HFD-induced MASLD mice model. Elevated serum ALT and AST activities are commonly used indicators of liver injury. As shown in Figures 6E,F, serum ALT and AST activities were significantly higher in the HFD group than in the Control group, indicating that HFD induced liver injury, while supplementation with PIO and PA alleviated the impaired liver function. The further results on related substances in the oxidation pathway showed that the levels of and malondialdehyde (MDA, *p* < 0.0001, Figure 6G) in the PA and PIO groups were lower than those in the model group, while the superoxide dismutase (SOD, *p* < 0.0001, Figure 6H) levels were higher than those in the model group. The above results indicate that PA has the effect of antioxidants on the mice liver tissue.

### 3.5 Effects of PA on FFA-treated BRL-3A cells *in vitro*

**Figure 7:**
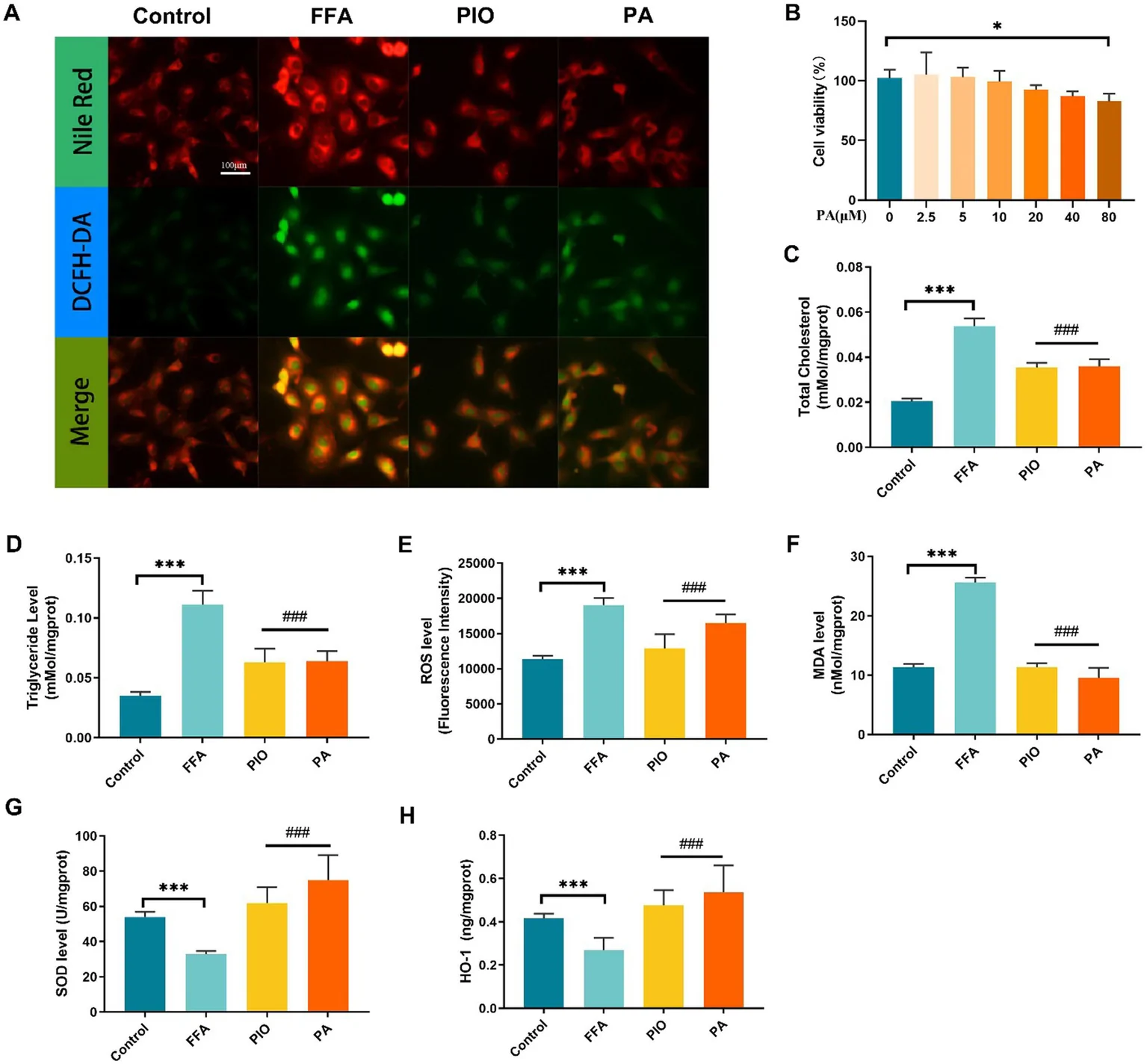
Effects of PA on FFA-treated BRL-3A cells (*n* = 3). **(A)** Signal quantification of BRL-3A cells treated with 1 mM FFA and stained with Nile red and DCFH-DA. **(B)** CCK-8 assay evaluating PA cytotoxicity. **(C)** Total cholesterol (TC; mmol/mg protein) levels. **(D)** Triglyceride (TG; mmol/mg protein) levels. **(E)** *ROS* levels, quantified by fluorescence intensity. **(F)** *MDA* (nmol/mg protein) levels. **(G)** *SOD* activity (U/mg protein). **(H)** *HO-1* (ng/mg protein) levels. Bars represent means ± SD. **p < 0.01 and ***p < 0.001 vs. control; ##*p* < 0.01 and ###*p* < 0.001 vs. model. Statistical significance was set at *p* < 0.05. Group means were compared using one-way ANOVA followed by Dunnett’s multiple-comparisons test. Experiments were performed in triplicate.

To further delineate the mechanisms by which PA modulates lipid metabolism and oxidative stress, we used FFA-treated BRL-3A cells as an in *vitro* model of MASLD. The pharmacological activity of PA was assessed by comparing model cells with and without PA treatment. The immortalized BRL-3A cell line provided a robust and reproducible platform for our initial mechanistic studies of hepatic steatosis and associated oxidative stress. CCK-8 assays showed that PA adversely affected cell viability at concentrations ≥ 40 μM. We chose a concentration of 20 μM for subsequent experiments, as it showed higher efficacy than other concentrations (Figure 7B).

Nile red staining revealed that PA and PIO treatments markedly attenuated the FFA-induced increase in lipid accumulation (Figure 7A). Consistent with the *in vivo* results, intracellular TC (*p* = 0.0004) and TG (*p* = 0.0005) levels in the PA group were significantly lower¬ than in the model group (Figures 7C,D). DCFH-DA staining showed significantly higher fluorescence intensity in the model group than in all other groups. Furthermore, ROS (*p* = 0.0003) and MDA (*p* < 0.0001) levels were significantly reduced (Figures 7E,F), whereas SOD activity (*p* < 0.0001) and HO-1 expression (*p* < 0.0001) were significantly increased (Figures 7G,H) in the PA group relative to the model group. These coordinated changes demonstrate that PA exerts potent antioxidant effects comparable to those of PIO. The lipid-modulating and antioxidant activities of PA were consistently observed in both in *vivo* and *in vitro* models.

### 3.6 PA promotes NRF2 nuclear translocation

**Figure 8:**
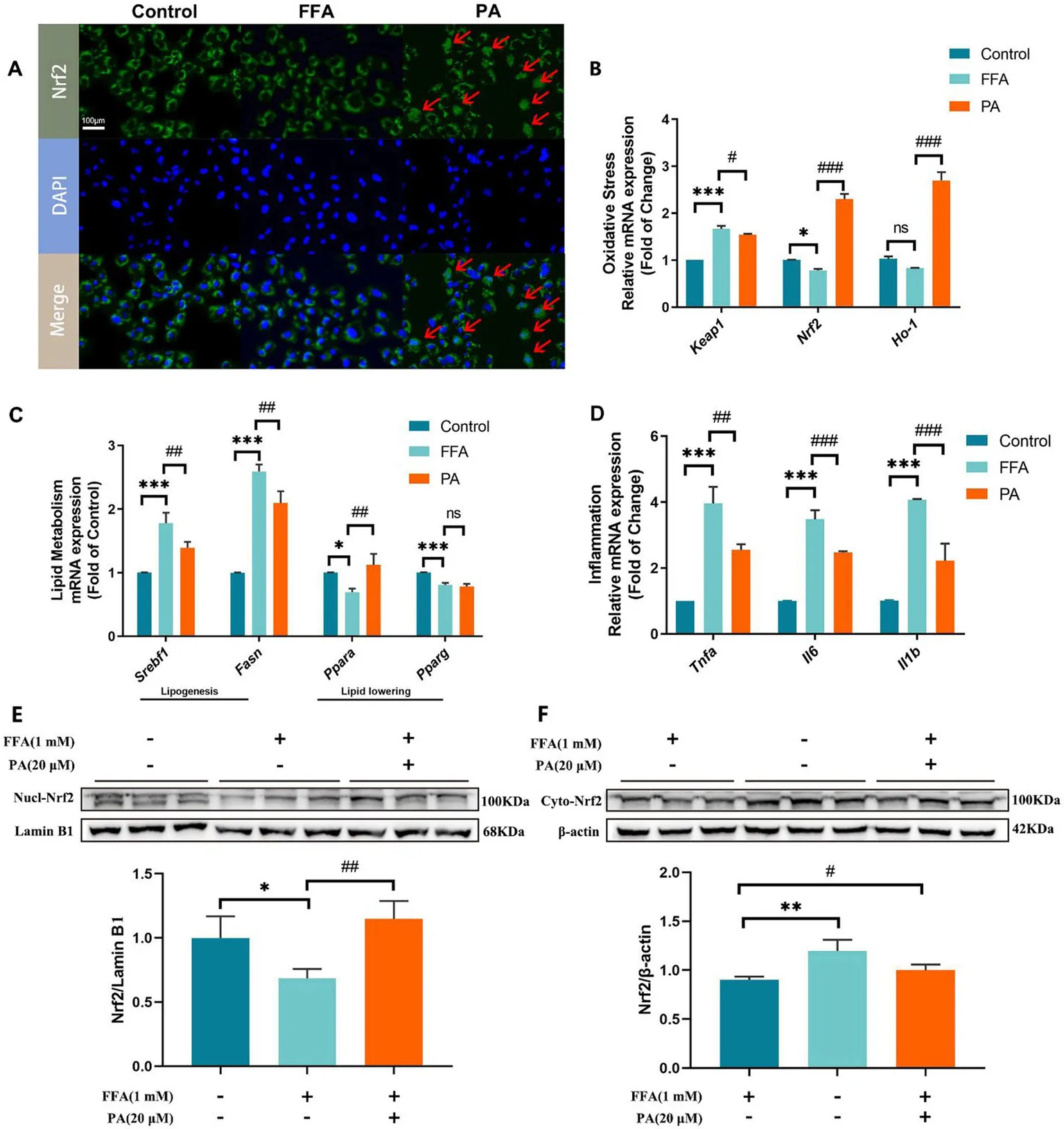
Effects of PA on mRNA and protein expression in FFA-treated BRL-3A cells *in vitro* (*n* = 3). **(A)** Immunofluorescence quantification and localization of NRF2 in BRL-3A cells counterstained with DAPI. **(B)** Oxidative stress-related gene expression. **(C)** Lipid metabolism-related gene expression. **(D)** Inflammation-related gene expression. **(E)** Western blot analysis of nuclear NRF2 and lamin B1. **(F)** Western blot analysis of cytoplasmic NRF2 and *β*-actin. Bars represent means ± SD. n.s., not significant. *p < 0.05; **p < 0.01; ***p < 0.001 vs. control. #*p* < 0.05, ##*p* < 0.01 and ###*p* < 0.001 vs. FFA. Statistical significance was set at *p* < 0.05. Group means were compared using one-way ANOVA followed by Dunnett’s multiple-comparisons test. Experiments were performed in triplicate.

This work used immunofluorescence staining and RT-qPCR to investigate the intrinsic mechanisms in FFA-treated BRL-3A cells. The nucleus was stained by DAPI, so that we could visually observe whether NRF2 was in the cytoplasm or the nucleus. Immunofluorescence staining indicated that NRF2 was predominantly localized in the nucleus in the PA group, whereas it was mainly in the cytoplasm in the control and model groups (Figure 8A), indicating a significant nuclear translocation following PA treatment. PA modulated the Keap1–Nrf2 signaling pathway by downregulating *Keap1* (*p* = 0.0159) and upregulating *Nrf2* (*p* = 0.0135) and its downstream target *Ho-1* (*p* < 0.0001; Figure 8B). PA also affected genes involved in lipid metabolism (Figure 8C), and significantly downregulated the mRNA expression of pro-inflammatory cytokines *Tnfa* (*p* = 0.0024), *Il6* (*p* = 0.0005), and *Il1b* (*p* = 0.0005; Figure 8D). PA increased the total cellular level of NRF2 (nuclear and cytoplasmic fractions combined; Figures 8E,F).

### 3.7 The lipid droplet-reducing effect of PA in BRL-3A cells is NRF2-dependent

**Figure 9:**
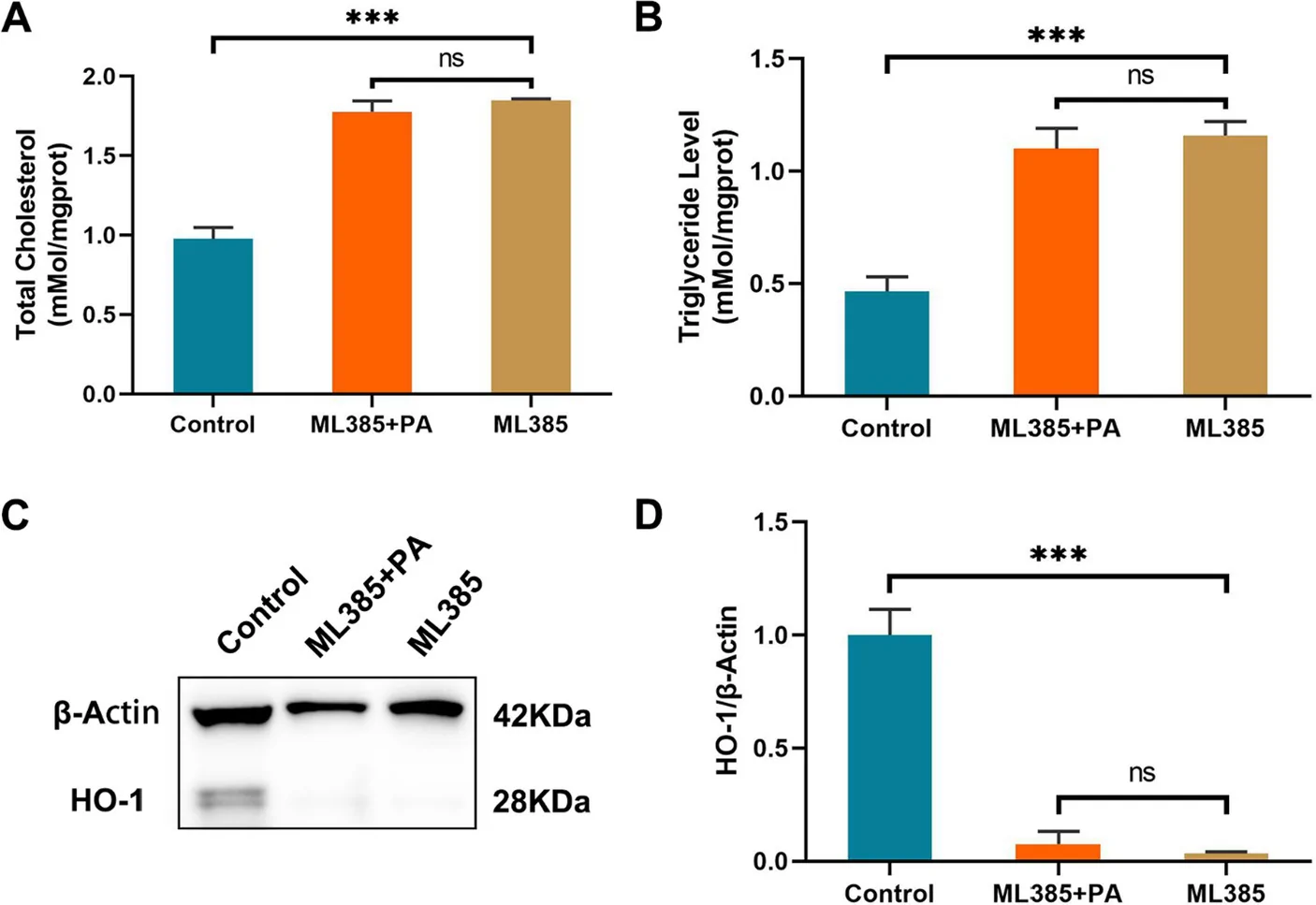
The lipid droplet-lowering effect of PA in BRL-3A cells is NRF2-dependent. **(A, B)** Cellular TC and TG levels. **(C, D)** Western blot analysis and quantification of HO-1 expression normalized to β-actin (*n* = 3). Bars represent means ± SD. n.s., not significant. ***p < 0.001. Statistical significance was set at *p* < 0.05. Group means were compared using one-way ANOVA followed by Dunnett’s multiple-comparisons test. Experiments were performed in triplicate.

To further verify whether PA exerts its effects through NRF2, we examined the impact of ML385 on PA efficacy in BRL-3A cells. The TC- and TG-lowering effects of PA were markedly attenuated in the presence of ML-385 (Figures 9A,B). Western blot analysis showed that under PA treatment, ML-385 did not significantly increase downstream HO-1 protein expression (Figures 9C,D).

## 4 Discussion

MASLD encompasses a broad spectrum of liver disorders strongly associated with dysmetabolic diseases, such as obesity, insulin resistance, and type 2 diabetes. Its pathogenesis involves complex interactions among lipid metabolic dysregulation, oxidative stress, inflammation, and hepatocellular injury (29). To our knowledge, no satisfactory strategy currently exists to inhibit MASLD. In this context, active ingredients derived from traditional Chinese medicine (TCM) have attracted increasing attention as potential therapeutic candidates (30). PA is a lanost-8-ene-type triterpene compound found in *Poria cocos* (31). Multiple pharmacological studies have demonstrated that *Poria cocos* extracts exert favorable lipid-lowering effects and ameliorate hepatic inflammation (32, 33). Studies have documented the role of PA in suppressing mouse ear swelling induced by 12-o-tetradecanoylphorbol-13-acetate (17). However, the mechanisms by which it affects other antioxidant pathways remain unclear. The present study extends these findings by showing that PA attenuates hepatic steatosis and oxidative injury, potentially through the Keap1–Nrf2–HO-1 pathway.

The “multiple-hit” theory has been employed to describe the complex pathogenesis of MASLD. Abnormal lipid metabolism initiates hepatic steatosis. This study used HCD-induced zebrafish, HFD-fed mice, and FFA-treated BRL-3A cells, and revealed that PA lowered TC and TG levels and inhibited *srebf1* and *fasn* expression. These findings suggest that PA may alleviate steatosis not only by reducing lipid accumulation but also by suppressing hepatic lipogenic transcription. This mechanism complements previous reports showing that PA alleviates hepatic steatosis by activating the FGF21/FGFR1 signaling axis, and inhibiting p38 MAPK activation (34). Together, these studies support the view that PA restores lipid homeostasis through multiple coordinated mechanisms.

Oxidative stress is a central link between lipid overload and hepatocellular injury in MASLD (35). Excess FFAs promote ROS generation, lipid peroxidation, mitochondrial dysfunction, and inflammatory responses (22). In the present study, PA reduced ROS and MDA levels, restored SOD activity, and decreased serum ALT and AST activities, indicating attenuation of oxidative damage and liver injury. Consistent with previous evidence that PA improves oxidative stress-associated lipid metabolic disorders through SIRT6-related pathways (36), and suppresses lipid peroxidation and ferroptosis through PPARα signaling (19). However, unlike these studies, our work focused on the Keap1–Nrf2–HO-1 pathway, which represents a canonical intracellular antioxidant defense system.

Nrf2 has been a pivotal therapeutic target in studies on antioxidant effects (37). Under non-oxidative stress conditions, Keap1 promotes NRF2 ubiquitination and degradation (38). To elucidate the mechanism of PA, the expression of genes associated with oxidative stress was measured in zebrafish and BRL-3A cells. RT-qPCR showed that PA attenuated ROS-driven Keap1 overexpression while increasing Nrf2 and Ho-1 expression, PA enhanced antioxidative activity. Upon oxidative stress, the cysteine residues of Keap1 can bind ROS at high concentrations, leading to conformational changes in Keap1 (35) and its dissociation from Nrf2. Once free, Nrf2 translocates to the nucleus, binds to antioxidant response elements, and initiates the expression of HO-1 by activating a downstream antioxidant gene (39). The abundantly expressed HO-1 exerts an antioxidant effect and reduces intracellular ROS (40). Further western blot and immunofluorescence assays showed that PA facilitated NRF2 nuclear translocation, suggesting that the NRF2 pathway is a major target of PA. HO-1, a critical anti-oxidant effector downstream of the Keap1–Nrf2 axis, indirectly reflects the nuclear activity of NRF2. These findings suggest that part of PA’s antioxidative effect is mediated through the Keap1–Nrf2–HO-1 axis. PA’s target in this pathway might be a protein related to NRF2 translocation into the nucleus. It is also possible that PA upregulates HO-1 expression by inhibiting NRF2 ubiquitination and degradation. To validate the critical role of NRF2 in this study, its specific inhibitor ML-385 was used *in vitro*. The results showed that PA failed to alleviate FFA-induced lipid accumulation in the absence of NRF2. These findings highlight NRF2 as a key mediator of the therapeutic effects of PA in MASLD.

In this study, multiple models were used to verify the antioxidant effect of PA. The zebrafish is a tractable vertebrate model for lipid metabolism research. Humans and zebrafish share a number of similar features relevant to MASLD. Moreover, zebrafish possesses additional advantages, such as rapid external development, optical transparency for real-time monitoring, and suitability for large-scale *in vivo* drug screening (41). However, given the limited organ complexity of zebrafish, the progression to more severe steatohepatitis involving inflammation and fibrosis cannot be studied in this model. Therefore, we additionally employed a HFD-treated mice model. Although we found that the Keap1–Nrf2–HO-1 axis may be a key mechanistic pathway underlying the anti-MASLD activity of PA, no direct mechanistic experiments have been conducted to confirm this hypothesis. In follow-up studies, we plan to use omics technologies or siRNA/CRISPR gene knockdown techniques to further verify this pathway in vivo.

## 5 Conclusion

In summary, PA ameliorated MASLD by mitigating lipid metabolic dysregulation and exerting antioxidant and anti-inflammatory effects in the HCD-induced zebrafish larval model, HFD-treated mice and the FFA-treated BRL-3A cell model. We showed that the Keap1–Nrf2–HO-1 axis might be a key mechanistic pathway through which PA exerts its anti-MASLD activity. However, no direct mechanistic experiments were conducted to confirm this hypothesis. This work suggests that PA exerts pharmacological effects relevant to MASLD, highlighting its potential as an anti-NAFLD/MASLD therapeutic agent and functional food ingredient.

## Data Availability

The datasets presented in this study can be found in online repositories. The names of the repository/repositories and accession number(s) can be found in the article/Supplementary material.

## Glossary

ANOVA: One-way analysis of variance
DCF: 2′,7-dichlorofluorescein
DCFH-DA: dichlorodihy-drofluorescein diacetate
dpf: days post fertilization
fasn: fatty acid synthase
FBS: free fatty acid
FFA: free fatty acid
HCD: high cholesterol diet
HE: hematoxylin–eosin
HO-1: heme oxygenase-1
IL-1β: interleukin 1β
IL-6: interleukin 6
Keap1: kelch-like ECH-associated protein 1
MASLD: metabolic dysfunction-associated steatotic liver disease
MDA: malondialdehyde; mmp9, matrix metalloproteinase
NAFLD: Nonalcoholic fatty liver disease
Nrf2: nuclear factor-like 2
ORO: oil red O
PA: Pachymic acid
PIO: Pioglitazone
PLA2: phospholipase A2
ppara: peroxisome proliferator-activated receptor alpha
Pparg: peroxisome proliferator-activated receptor gamma
ROS: reactive oxygen species
SOD: superoxide dismutase
srebf1: sterol-regulatory element binding proteins
TC: total cholesterol
TG: triglyceride
Tgfb1: transforming growth factor β1
TNF-*α*,: tumor necrosis factor alpha
