# Supplementary material for: Pachymic acid alleviates hepatic lipid accumulation and oxidative stress in MASLD via regulation of the Keap1–Nrf2–HO-1 signaling axis
Source: Front Nutr. 2026 Aug 18;13:1887176. doi: 10.3389/fnut.2026.1887176 (PMC13530000; doi:10.3389/fnut.2026.1887176)
Supplement: Supplementary file 1 [file Table_1.docx]

Supplementary Material

**Table S1.** Specific sequences of primers used in RT-qPCR.

| **Gene name** | **Species** | **Forward primer (5’->3’)** | **Reverse primer (5’->3’)** |
| --- | --- | --- | --- |
| ***srebf1*** | ***Danio rerio*** | CATCCACATGGCTCTGAGTG | CTCATCCACAAAGAAGCGGT |
| ***fasn*** | ***Danio rerio*** | ATCTGTTCCTGTTCGATGGC | AGCATATCTCGGCTGACGTT |
| ***pparab*** | ***Danio rerio*** | CGTCGTCAGGTGTTTACGGT | AGGCACTTCTGGAATCGACA |
| ***pparg*** | ***Danio rerio*** | CTGCCGCATACACAAGAAGA | TCACGTCACTGGAGAACTCG |
| ***tnfa*** | ***Danio rerio*** | GCTTATGAGCCATGCAGTGA | TGCCCAGTCTGTCTCCTTCT |
| ***il1b*** | ***Danio rerio*** | TGGCGAACGTCATCCAAG | GGAGCACTGGGCGACGCATA |
| ***il6*** | ***Danio rerio*** | AGACCGCTGCCTGTCTAAAA | TTTGATGTCGTTCACCAGGA |
| ***keap1*** | ***Danio rerio*** | CCAACGGCATAGAGGTAGTTAT | CCTGTATGTGGTAGGAGGGTT |
| ***nrf2*** | ***Danio rerio*** | TTGTCTTTGGTGAACGGAGGT | CTCGGAGGAGATGGAAGGAAG |
| ***ho-1*** | ***Danio rerio*** | GCTCAACATCCAGCTCTTTGAGG | GACAAAGTTCATGGCCCTGGGA |
| ***Tnf-α*** | ***Rattus norvegicus*** | CCTCTTCTCATTCCTGCTC | CTTCTCCTCCTTGTTGGG |
| ***Il-6*** | ***Rattus norvegicus*** | AGTTGCCTTCTTGGGACTGATGT | GGTCTGTTGTGGGTGGTATCCTC |
| ***Il-1β*** | ***Rattus norvegicus*** | TGCACCAGCTGCACATCACA | GCAGCGGTCAGGTGGATCTC |
| ***Srebf1*** | ***Rattus norvegicus*** | ACTGCTGTAAAGATGTACCCGTCCG | GGCACTGGCTCCTCTTTGATTCC |
| ***Fasn*** | ***Rattus norvegicus*** | CTTTGTGAGCCTCACCGCCAT | ATGCCATCAGGTTTCAGCCCC |
| ***Pparab*** | ***Rattus norvegicus*** | GCATGGCTGAGAAGACGCTTG | GGATAGCCTTGGCAAATTCCG |
| ***Pparg*** | ***Rattus norvegicus*** | TCCCGTTCACAAGAGCTGAC | ATAATAAGGCGGGGACGCAG |
| ***Keap1*** | ***Rattus norvegicus*** | TGCTCAACCGCTTGCTGTATG | CCAAGTGCTTCAGCAGGTACA |
| ***Nrf2*** | ***Rattus norvegicus*** | TTGTAGATGACCATGAGTCGC | TGTCCTGCTGTATGCTGCTT |
| ***Ho-1*** | ***Rattus norvegicus*** | GTAAATGCAGTGTTGGCCCC | ATGTGCCAGGCATCTCCTTC |
